# Optimal Achievable Transmission Capacity Scheme for Full-Duplex Multihop Wireless Networks

**DOI:** 10.3390/s22207849

**Published:** 2022-10-16

**Authors:** Aung Thura Phyo Khun, Yuto Lim, Yasuo Tan

**Affiliations:** Graduate School of Advanced Science and Technology, Japan Advanced Institute of Science and Technology, 1-1 Asahidai, Nomi City 923-1292, Ishikawa, Japan

**Keywords:** full-duplex system, capacity optimization, interference mitigation, transmit power control, multihop wireless network

## Abstract

Full-duplex (FD) communication has been attractive as the breakthrough technology for improving attainable spectral efficiency since the 5G mobile communication system. Previous research focused on self-interference cancellation and medium access control (MAC) protocol to realize the FD system in wireless networks. This paper proposes an optimal achievable transmission capacity (OATC) scheme for capacity optimization in the FD multihop wireless networks. In this paper, the proposed OATC scheme considers the temporal reuse for spectral efficiency and the spatial reuse with transmit power control scheme for interference mitigation and capacity optimization. OATC scheme controls the transmit power to mitigate interference and optimizes the transmission capacity, which leads to the optimal achievable network capacity. We conduct the performance evaluation through numerical simulations and compare it with the existing FD MAC protocols. The numerical simulations reveal that considering only the concurrent transmissions in the FD system does not guarantee optimal transmission capacity. Moreover, the hybrid mechanism, including the sequential transmissions, is also crucial because of the interference problem. Besides, numerical simulation validates that the proposed OATC scheme accomplishes the optimal achievable network capacity with lower interference power and higher achievable throughput than the existing MAC protocols.

## 1. Introduction

Full-duplex (FD) is well-known as the potential communication technology to improve the spectral efficiency of the transmission for the wireless system. FD communication simultaneously allows the transmission and reception in a single frequency channel. Several research works have focused on realizing FD communication by proposing the protocols and designing the system in different approaches in the networks of wireless LAN and mobile wireless [1,2,3,4,5,6]. For example, refs. [1,2] proposed the MAC protocols to realize the FD communication using the carrier sense multiple access with collision avoidance (CSMA/CA) concepts. The FD MAC protocols with the primary hand-shaking mechanism of request-to-send (RTS)/clear-to-send (CTS) were proposed in [3,4]. In the ad hoc network, Rahman et al. proposed Adhoc-FDMAC using the distributed coordination function (DCF) MAC standard [5]. Marlali et al. proposed the synchronized contention window FD (S-CW FD) protocol for synchronization in enabling the FD transmission, which can operate in both ad hoc and infrastructure modes of WLAN [6].

Unlike half-duplex (HD) communication, FD communication benefits in throughput gain, collision avoidance, hidden terminal problem reduction, and end-to-end delay mitigation in the wireless system. However, the concurrent transmissions in the FD system could bring possible increased traffic, higher interference, degraded link reliability, higher packet loss rate, and synchronization issues [7]. Therefore, there are several challenges in implementing FD communication in the wireless system. One of the challenges is the strong self-interference (SI) which is the signal disturbed from its own transmitting antenna to the receiving antenna of the FD-enabled wireless node. For this purpose, the researchers have been working on self-interference cancellation (SIC) techniques from different perspectives. The recent advances in antenna designs, signal processing techniques, and cancellation techniques can suppress up to 80–110 dB of SIC until the noise level of the transmission [8,9]. As a result, theoretically, FD communication has the potential to double the transmission capacity compared to HD communication [10]. In addition to SI from its own transmission, when multiple concurrent transmissions are taking place in the near vicinity over the same frequency channel, the FD system also has to deal with the inter-user interference (IUI), a.k.a. co-channel interference from other transmissions [11,12,13]. The authors in [14] claim that IUI affects the quality of the transmission signal, dominates the SI and results in network capacity degradation. Besides, ref. [15] described that controlling the cross-link interference can guarantee double capacity improvement in the FD system. Therefore, many researchers proposed resource allocation techniques, medium access control (MAC) techniques, and physical layer techniques as promising methods for mitigating IUI [16,17,18,19].

The ever-increasing capacity demand due to traffic growth in the limited spectrum of resources leads to the capacity optimization problem in wireless communication. As one of the critical drivers to providing efficient transmission for the future wireless system, it is worth exploring FD communication for capacity optimization. Since the multiple concurrent transmissions share the wireless transmission medium, when the FD capability is enabled, the transmission capacity in the multihop wireless network is limited because of the interference problem. As a result, interference mitigation plays the main factor in capacity optimization. The authors in [11,14,20,21] claimed that the FD system requires well planning to outperform the HD system counterpart. Besides that, FD is more suitable in the low transmit power network scenario, and power control is an efficient mechanism for effectively managing the interference power [22]. However, the current power control schemes tend to reduce the transmission capacity regardless of the transmission scheme and lack consideration of the tradeoff between interference power and capacity gain of the transmission. Therefore, it motivates us to investigate the mechanism for capacity optimization by considering the influence of power control in both interference mitigation and capacity gain of the transmission.

This paper investigates capacity optimization by mitigating interference with the concept of temporal reuse and spatial reuse techniques in the FD system. We propose the optimal achievable transmission capacity (OATC) scheme for ensuring optimal achievable network capacity in FD multihop wireless networks. Unlike the previous interference mitigation and power control schemes, OATC minimizes the interference power and optimizes the transmission capacity through the transmission scheme to manage the concurrent transmissions and power control scheme to adjust the coverage area of the transmission. Therefore, our objective is to propose a novel OATC scheme for optimizing the transmission capacity with minimum interference power of each single FD transmission in the FD multihop wireless networks.

### 1.1. Literature Review

Plentiful research works designed the MAC protocol to enable FD communication for capacity optimization in different perspectives [9]. Amin and Hossain designed the distributed MAC protocol with transmit power control based on the RTS/CTS mechanism for the FD wireless local network [3]. The authors argued that transmit power control is an efficient mechanism. They applied it to allow the secondary transceiver to adjust the carrier sensing range and reduce the inter-station interference. In their research, the proposed protocol can achieve up to 41% throughput improvement compared to the existing FD MAC protocols. In prior work, Song et al. proposed the CSMA/CA-based FD MAC protocol with the cut-through mechanism [2]. The protocol allows the FD nodes to detect collisions during the transmission and terminate transmitting if the collision is detected. In their research, the proposed protocol can improve the saturation throughput by more than twice the HD MAC protocol. Similar research work with CSMA/CA-based FD MAC protocol in [23], Kim et al. considered user scheduling for further capacity improvement. Their simulation results show a significant throughput gain than the loss due to collision because of high transmission probability even when the number of users increases in the network. In addition, in the hybrid approach with FD/HD system, the research in [24] analyzed the system throughput with adaptive communication mode of FD and HD based on the interference strengths of the FD wireless nodes.

On the other hand, plenty of research has focused on capacity optimization with interference mitigation in the FD system in both wireless LAN and mobile wireless networks. For example, in mobile wireless, Tang et al. derived capacity optimization by cancelling IUI from other transmissions [17]. The simulation result shows that the perfect cancellation can bring more than 60% capacity gain even in a dense network environment. In similar research on mobile wireless, Wu et al. proposed the IUI suppression scheme by transmitting the additional reversed signal at the base station [16]. However, transmitting the additional signal can cause further energy-consuming. Therefore, as the different perspectives to approach the interference mitigation problem, the transmit power control (TPC) scheme is considered to mitigate IUI [19]. For example, Qu et al. proposed the multiuser FD MAC protocol, which considers the power control to manage the uplink for interference [25]. In their research, the simulation result shows that the lower transmit power that satisfies the signal-to-interference-plus-noise ratio (SINR) threshold can bring a better network capacity. Another relevant research work is [18], which proposed the limited area-based interference control scheme in mobile wireless using TPC. In addition, Samy et al. designed the transmit power optimization problem to maximize the sum-rate and received SINR in the wireless relay network [26]. Furthermore, Kai et al. presented the joint user scheduling and power control scheme as the performance optimization in the wireless LAN [27]. In their simulation results, the proposed scheme brings up to 80% of capacity improvement compared to the HD system. In [28], the use-up-link-capacity iterative algorithm formulates the throughput optimization, which pairs the transmission with binary scheduling variables in FD cellular network. Similar to [14], the proposed distributed joint user scheduling and power allocation achieved nearly twice the capacity improvement in an indoor multicell network.

The existing FD MAC protocols only consider concurrent transmissions with temporal reuse for capacity optimization in the wireless network. Even in the hybrid approach with FD/HD system, the MAC protocol only considers the concurrent transmission (CT) scheme in FD mode and the sequential transmission scheme in HD mode. With the motivation of lack to consider both the concurrent transmission and sequential transmission in the FD system, we proposed a mixture of concurrent and sequential transmission (MCST) scheme for capacity optimization in the FD multihop wireless networks by considering both the advantages of concurrent transmission and sequential transmission in [29]. However, we lack to consider the tradeoff between the interference power and capacity gain of the transmission. In [30], we also studied the consensus transmit power control (CTPC) scheme for capacity optimization through power control in the FD multihop wireless networks and elucidated that the CTPC with optimal consensus coefficient value improves the throughput with the minimum transmit power and interference power. Therefore, the reviewed literature shows that FD communication is worth studying and still needs investigation for capacity optimization in future advanced wireless networks.

### 1.2. Contributions

This paper investigates capacity optimization in the FD multihop wireless networks with the aid of the relay node. The contributions of this paper are summarized as follows.

- This paper contributes that the findings of the CT scheme only and the MCST scheme only cannot provide the best achievable network capacity all the time, even with the aid of transmit power control scheme in the FD multihop wireless networks. As a result, the OATC scheme is proposed to combine these two schemes to achieve the optimal achievable capacity.
- This paper introduces an algorithm for the OATC scheme to choose either the CT scheme or MCST scheme by using the tradeoff threshold, which is the tradeoff to consider both the interference power and the received power of the ongoing transmission.
- Through numerical simulation results, this paper reveals that the proposed OATC scheme not only applies the high ratio of the CT scheme when the network is not dense to achieve the optimal achievable capacity but also reduces total interference power.

We note that [31] presented a part of the contributions to the OATC scheme as a preliminary study of saturation throughput analysis. However, we assumed the consensus coefficient is one as a constant in the CTPC algorithm. Therefore, compared to [31], the consensus coefficient impact is considered in this paper for a complete OATC scheme of the considered research problem.

The organization of the paper is as follows. Section 2 presents the system model, and Section 3 introduces the proposed OATC scheme. Section 4 presents the numerical simulation scenarios and settings, evaluation metrics, results, and discussion. Finally, Section 5 concludes the paper.

## 2. System Model

**Figure 1:**
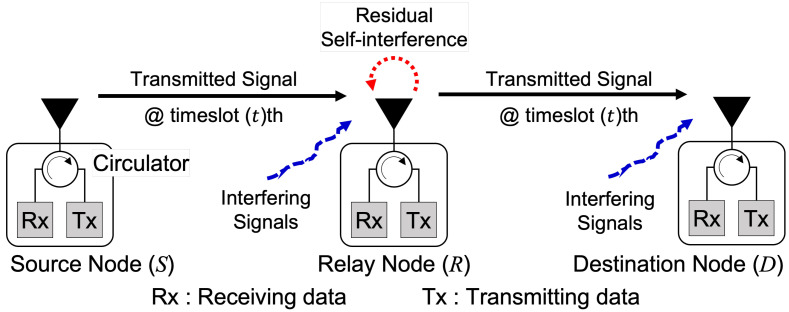
Shared-antenna full-duplex system with single relaying transmission flow.

This section presents the system model with the distributed wireless network without the central control nodes. In the network, we consider a single relaying transmission flow in which a source node (*S*) communicates to a destination node (*D*) with the help of a relay node (*R*), as illustrated in Figure 1. We assume that all the wireless nodes are enabled as a shared-antenna FD system [32]. They can transmit and receive simultaneously in a single frequency band through the use of a circulator. In the wireless system, the FD-enabled relay node adopts the SIC technique to suppress SI. Therefore, we assume SI can be suppressed until the noise level and omit the effect of SI as in [17]. The FD relaying system can apply the decode-and-forward and amplify-and-forward relaying strategies. However, we assume this research applies no relaying strategies for simplicity. In this work, we also assume the relay node knows the perfect channel state information (CSI) from other nodes. Hence, we define the basic relaying flow (BRF) transmission as the combination of source-relay (SR) transmission and relay-destination (RD) transmission by omitting the time synchronization problem. In addition, due to the shadowing effects, we assume that there is no direct transmission link from *S* to *D* as in [33]. Let N={1,⋯,n,⋯,N} denote the set of nodes where *N* is the total number of nodes. Each BRF transmission can be selected from the set of BRF transmission M={1,⋯,m,⋯,M} where *M* is the number of BRF transmissions and M=N/3. In this research, to consider the tradeoff between the total interference power level and the achievable network capacity in the presence of TPC, the selection of node combinations in a single BRF transmission is random for the theoretical analysis purpose in the single network.

### 2.1. Channel Model

The attenuation of the transmission link over a communication channel depends on the signal propagation model of the Log-distance pathloss model. With the Friis free space model, the channel gain in the unit of decibel [dB] at the receiving node *j* from the transmitting node *i*, where i,j∈N is defined as

(1) $$PL_{ij}=PL_{0}+10\cdot \alpha \cdot log_{10}\left(\frac{d_{ij}}{d_{0}}\right)-W_{ij}+X_{\sigma }$$

where $PL_{0}=20\cdot log_{10}\left(d_{0}\right)$, α is the attenuation constant, $d_{0}$ is the decorrelation distance which is 1 m, $d_{ij}$ is the euclidean distance between node *i* and node *j*, $W_{ij}$ is the wall attenuation and $X_{\sigma }$ is the additive and Gaussian random variable with zero mean and standard deviation of σ. Then, the received power ratio between transmitting node *i* and receiving node *j* is

(2) $$G_{ij}=\frac{1}{10^{\left(\frac{PL_{ij}}{10}\right)}}$$

### 2.2. SINR Model

A successful transmission occurs only if the SINR is above a certain threshold. The SINR obtained at the receiving node *j* from the transmitting node *i* is computed as

(3) $$SINR_{ij}=\frac{G_{ij}\cdot P_{i}}{\eta_{j}\cdot B+\sum_{k\in I,k\neq i}G_{kj}\cdot P_{k}}$$

where $P_{i}$ is the transmit power of the node *i*, $\eta_{j}$ is the noise level at node *j*, *B* is the channel bandwidth, and I is the set of interfering nodes to the ongoing transmission from node *i* to node *j*. SNR-based interference model is assumed to compute I when there are concurrent transmissions in the vicinity of the transmission medium [29].

An interference model determines the interfering nodes according to the received signal-to-noise ratio (SNR) of the ongoing transmission from node *i* to node *j* and is computed as

(4) $$SNR_{ij}=\frac{G_{ij}\cdot P_{i}}{\eta_{j}\cdot B}$$

For example, if node *k* is the concurrent transmitting node in the same transmission medium, the SNR of node *k* to transmitting node *i* and receiving node *j* of the ongoing transmission is $SNR_{ki}$ and $SNR_{kj}$, respectively. Then, if $SNR_{ki}$ and/or $SNR_{kj}$ is less than $SNR_{ij}$, the transmitting node *k* is called interfering node and k∈I.

### 2.3. Link Capacity Model

Using the Shannon theorem [34], the transmission rate [bit/s] over a communication channel for the ongoing transmission from node *i* to node *j* is defined as

(5) $$R_{ij}=B\cdot log_{2}\left(1+SINR_{ij}\right)$$

### 2.4. Achievable Single BRF Transmission Capacity

**Figure 2:**
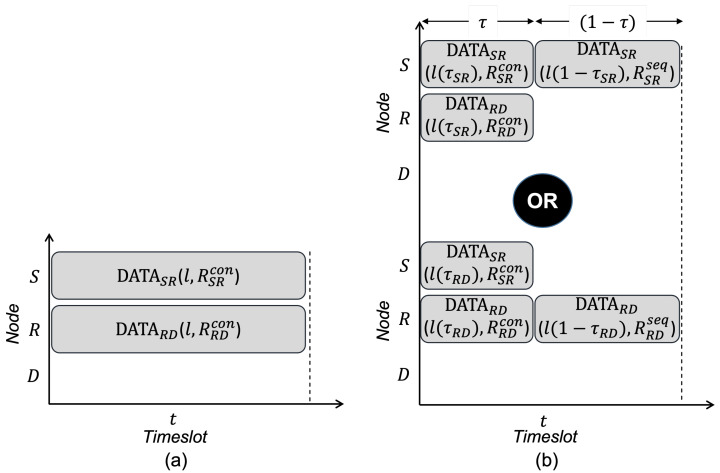
Transmission scheme in Full-duplex system with single relaying transmission flow: (**a**) concurrent transmission scheme and (**b**) mixture of concurrent and sequential transmission scheme.

In the FD system, several transmissions can proceed simultaneously in the single time/frequency band through the CT scheme. As shown in Figure 1, the relay node *R* can receive the transmission from the source node *S* and proceed to the destination node *D* simultaneously. Figure 2a shows an example of a CT scheme for a BRF transmission, and the achievable transmission capacity is computed as

(6) $$C=R_{SR}^{con}+R_{RD}^{con}$$

where $R_{SR}^{con}$ and $R_{RD}^{con}$ are the transmission rates $R_{SR}$ and $R_{RD}$ based on $SINR_{SR}$ of SR transmission and $SINR_{RD}$ of RD transmission, respectively.

Through the cooperative MCST scheme, the achievable transmission capacity of a BRF transmission can be maximized as presented in [29]. MCST performs the concurrent transmissions in the time fraction τ of the timeslot and sequential transmission in the remaining time of the timeslot. MCST optimizes the achievable transmission capacity by selecting the time fraction τ and the sequential transmission as shown in Figure 2b. The time fraction τ can be computed as the following equation

$$\tau =\left\{\begin{array}{c} \frac{R_{SR}^{seq}}{R_{SR}^{seq}-R_{SR}^{con}+R_{RD}^{con}}\;\mathrm{as}\;\tau_{SR}\;\mathrm{if}\;SR\;\mathrm{is}\;\mathrm{sequential}\;\mathrm{transmission}\\ \frac{R_{RD}^{seq}}{R_{RD}^{seq}-R_{RD}^{con}+R_{SR}^{con}}\;\mathrm{as}\;\tau_{RD}\;\mathrm{if}\;RD\;\mathrm{is}\;\mathrm{sequential}\;\mathrm{transmission} \end{array}\right.$$

where $R_{SR}^{seq}$ and $R_{RD}^{seq}$ are the transmission rates $R_{SR}$ and $R_{RD}$ based on $SNR_{SR}$ of SR transmission and $SNR_{RD}$ of RD transmission, respectively. Then, the achievable transmission capacity of a BRF transmission is computed by choosing the sequential transmission, which gives the optimal transmission capacity as the following

(7) $$\begin{array}{cc} C=\max\{(\tau_{SR}\cdot \left(R_{SR}^{con}+R_{RD}^{con}\right)+\left(1-\tau_{SR}\right) & \cdot R_{SR}^{seq}),\\ (\tau_{RD}\cdot & \left(R_{SR}^{con}+R_{RD}^{con}\right)+\left(1-\tau_{RD}\right)\cdot R_{RD}^{seq})\} \end{array}$$

where $\tau_{SR}$ and $\tau_{RD}$ are the τ values as SR transmission and RD transmission are the choice of sequential transmissions, respectively.

### 2.5. Transmit Power Control Scheme

We obtain the achievable transmission capacity from the previous section. Hereafter, the TPC scheme is applied to advance the transmission capacity while mitigating the interference, subjective to the minimum transmit power of the nodes. We apply the CTPC scheme in this research work among the existing TPC schemes. CTPC converges all the transmitting nodes to an average transmission rate by adjusting the transmit power of each node [30,35]. In CTPC, the algorithm maximizes the minimum transmission rate of the ongoing transmissions by adjusting the transmit power. As a result, all transmission rates converge on the average transmission rate of all existing BRF transmissions, which optimizes the achievable network capacity. Therefore, we aim to attempt the capacity optimization of the transmissions while satisfying the SINR constraints, and the optimization problem for TPC is formulated as

(8) $$\begin{array}{cc} \; & F=\max_{P_{i}}\;\mathrm{mean}\left\{\forall R_{ij}\right\}\\ \; & A=\max_{P_{i}}\;\mathrm{mean}\left\{\forall F\right\}\times C\\ \; & \mathrm{subject}\mathrm{to}\;0\leq P_{i}\leq P_{max}\;,\;i\in N\\ \; & \;\;\;\;\;SINR_{ij}\geq \beta \;,\;i,j\in N \end{array}$$

where $R_{ij}$ is the transmission rate from node *i* to node *j* of a BRF transmission, *F* is the minimum link rate of each BRF transmission, C is the consensus coefficient that depends on the node-to-node distance of the network topology, *A* is the average link rate of all BRF transmissions, $P_{i}$ is the transmit power of node *i*, $P_{max}$ is the transmit power budget, and β is the SINR threshold.

## 3. Optimal Achievable Transmission Capacity Scheme

This section describes an optimal achievable transmission capacity (OATC) scheme to optimize the achievable transmission capacity of the BRF transmission in the FD multihop wireless networks. By applying the temporal reuse mechanism, OATC allows the relay node cooperatively select the CT scheme or MCST scheme to achieve the optimal achievable transmission capacity of the BRF transmission. By applying the hybrid scheduling policy of [14], OATC applies the MCST scheme, which gives its advantage of capacity gain. Otherwise, it applies the default CT scheme for BRF transmission. Besides, OATC integrates with the TPC mechanism to mitigate the IUI of the concurrent transmissions and accomplishes the objective function of achievable transmission capacity while satisfying the SINR threshold. The existing transmission schemes tend to achieve the optimal achievable transmission capacity without considering the tradeoff between interference power and capacity gain of the transmission. Therefore, the achievable transmission capacity is still limited when the network becomes dense. OATC attempts to overcome the interference issues and optimize the achievable transmission capacity by integrating the temporal and spatial reuse mechanisms.

The proposed OATC scheme attains the optimal achievable transmission capacity under the transmit power constraint with three main steps. The first step computes the transmission rate of each ongoing transmission through the CT scheme and MCST scheme. The second step applies the CTPC scheme to the CT and MCST scheme to obtain the appropriate transmit power for optimizing the achievable transmission capacity while mitigating the total interference power. Third, the operational transmission scheme, either the CT scheme or MCST scheme with TPC, is determined according to the defined tradeoff threshold. The tradeoff threshold T is the ratio of interference power to the received power of the receiving node in the BRF transmission and computed as

(9) $$T=\frac{I^{*}}{G^{*}}$$

where $I^{*}=\sum_{k\in I,k\neq i}G_{kj}\cdot P_{k}$ is the interference power and $G^{*}=G_{ij}\cdot P_{i}$ is the received power of the ongoing transmission. OATC selects the operational transmission scheme with the lower T for higher achievable transmission capacity. The lower tradeoff threshold gives the lower interference power and the higher achievable transmission capacity, which optimizes the achievable network capacity of the entire network.

OATC scheme works cooperatively according to the three algorithms executed in the relay node by using the simple compute-and-compare mechanism as in [36]. As illustrated in Algorithm 1, CT with CTPC computes the required transmit power, transmission rate of each transmitting node as the choice transmission rate R, and its threshold for the BRF transmissions through the CT scheme. In the algorithm, step 2 computes the transmission rate through the CT scheme from the SINR received. And then, CTPC is applied to optimize the achievable transmission capacity. Algorithm 2 describes the operation of MCST with CTPC. In the algorithm, step 2 to step 11 optimally chooses the transmission rate of the ongoing transmission through the MCST scheme. And then, CTPC is applied to mitigate the interference power by satisfying the required threshold condition for successful transmission. Finally, Algorithm 3 determines the operational transmission scheme of either CT scheme or MCST scheme according to the T and results in the transmit power and the transmission rate for the transmission.
**Algorithm 1** CT with CTPC Algorithm**Definition:** $TC_{ct}\left(P_{ct},R_{ct},T_{ct}\right)$ is transmission control set, *P* is the transmit power and R is the choice transmission rate, T is the threshold of CT, *t* is timeslot, *i* is the transmitting node, *j* is the receiving node**Input:** $SNR_{SR}$, $SNR_{RD}$, $SINR_{SR}$, $SINR_{RD}$**Output:** $TC_{ct}\left(P_{ct},R_{ct},T_{ct}\right)$   **function** CT/CTPC    Calculate $R_{SR}^{con}\left(t\right)$, $R_{RD}^{con}\left(t\right)$    Calculate target rate of each flow F(t) where     $F\left(t\right)⇐mean\{R_{SR}^{con}\left(t\right),R_{RD}^{con}\left(t\right)\}$    Calculate next target rate of all flows A(t+1) where     A(t)⇐mean{∀F(t)}    A(t+1)=A(t)×C    **if** A(t+1)=F(t) **then**      $P_{i}\left(t+1\right)=P_{i}\left(t\right)$      Set t←t+1. Go to step 13    **else**      Calculate $P_{i}\left(t+1\right)$ from A(t+1)      Set t←t+1. Go to step 2    **end if**    Set $P_{ct}=P_{i}\left(t\right)$    Set $R_{ct}=R_{ij}^{con}\left(t\right)$    Calculate $T_{ct}$    **return** $P_{ct},R_{ct},T_{ct}$  **end function**

**Algorithm 2** MCST with CTPC Algorithm
**Definition:**  $TC_{mcst}\left(P_{mcst},R_{mcst},T_{mcst}\right)$ is transmission control set, *P* is the transmit power and R is choice transmission rate, T is the threshold of MCST, *t* is timeslot, *i* is the transmitting node
**Input:**  $SNR_{SR}$, $SNR_{RD}$, $SINR_{SR}$, $SINR_{RD}$**Output:** 

$TC_{mcst}\left(P_{mcst},R_{mcst},T_{mcst}\right)$

  1:  **function** MCST/CTPC2:     Calculate $R_{SR}^{seq}\left(t\right)$, $R_{RD}^{seq}\left(t\right)$, $R_{SR}^{con}\left(t\right)$, $R_{RD}^{con}\left(t\right)$3:     Compute $\tau_{SR}\left(t\right)=\frac{R_{SR}^{seq}\left(t\right)}{R_{SR}^{seq}\left(t\right)-R_{SR}^{con}\left(t\right)+R_{RD}^{con}\left(t\right)}$4:     Compute $\tau_{RD}\left(t\right)=\frac{R_{RD}^{seq}\left(t\right)}{R_{RD}^{seq}\left(t\right)-R_{RD}^{con}\left(t\right)+R_{SR}^{con}\left(t\right)}$5:     Compute $C_{SR}\left(t\right)=\tau_{SR}\left(t\right)\cdot \left(R_{SR}^{con}\left(t\right)+R_{RD}^{con}\left(t\right)\right)+\left(1-\tau_{SR}\left(t\right)\right)\cdot R_{SR}^{seq}\left(t\right)$6:     Compute $C_{RD}\left(t\right)=\tau_{RD}\left(t\right)\cdot \left(R_{SR}^{con}\left(t\right)+R_{RD}^{con}\left(t\right)\right)+\left(1-\tau_{RD}\left(t\right)\right)\cdot R_{RD}^{seq}\left(t\right)$7:      **if** $C_{SR}\left(t\right)>C_{RD}\left(t\right)$ **then**8:        Set $R\left(t\right)\leftarrow \tau_{SR}\cdot \left(R_{SR}^{con}\right)+\left(1-\tau_{SR}\right)\cdot R_{SR}^{seq}$9:      **else**10:         Set $R\left(t\right)\leftarrow \tau_{RD}\cdot \left(R_{RD}^{con}\right)+\left(1-\tau_{RD}\right)\cdot R_{RD}^{seq}$11:      **end if**12:      Calculate target rate of each flow F(t) where       F(t)⇐mean{R(t)}13:      Calculate next target rate of all flows A(t+1) where       A(t)⇐mean{∀F(t)}14:      A(t+1)=A(t)×C15:      **if** A(t+1)=F(t) **then**16:    $P_{i}\left(t+1\right)=P_{i}\left(t\right)$17:    Set t←t+1. Go to step 2218:      **else**19:    Calculate $P_{i}\left(t+1\right)$ from A(t+1)20:    Set t←t+1. Go to step 221:      **end if**22:      Set $P_{mcst}=P_{i}\left(t\right)$23:      Set $R_{mcst}=R\left(t\right)$24:      Calculate $T_{mcst}$25:      **return** $P_{mcst},R_{mcst},T_{mcst}$26:  **end function**


**Algorithm 3** OATC with CTPC Algorithm
**Input:**  $TC_{ct}\left(P_{ct},R_{ct},T_{ct}\right)$,

$TC_{mcst}\left(P_{mcst},R_{mcst},T_{mcst}\right)$

**Output:** *P* and R  1:
**function** OATC
2:   **if** $T_{mcst}\leq T_{ct}$ **then**3:          Apply MCST/CTPC4:          Set $P=P_{mcst}$, $R=R_{mcst}$5:   **else**6:          Apply CT/CTPC7:          Set $P=P_{ct}$, $R=R_{ct}$8:   **end if**9:     **return** P,R10:
**end function**



## 4. Numerical Simulations

This section evaluates the network performance of the proposed OATC scheme through simulations. The performance evaluation compares the existing FD MAC protocols with RTS/FCTS [4], which considers only the concurrent transmissions without any sequential transmissions, and FD-MCST [29] with both concurrent and sequential transmissions in the multihop wireless networks. Both existing FD MAC protocols are not considering the TPC scheme.

### 4.1. Simulation Scenarios and Settings

**Figure 3:**
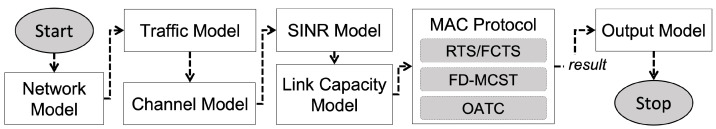
Block diagram of numerical analysis.

The simulation parameters and settings are listed in Table 1 with the standard specification of IEEE802.11ac [37]. In this paper, the simulation program is written using MATLAB R2021a. It consists of two scenarios: the influence on the DATA length in a 30-node random topology scenario and the influence on the network density with fixed DATA length. Figure 3 illustrates the block diagram of the numerical analysis. In the network model, a different network topology is randomly generated with a uniform distribution of the node allocation in the network coverage area. As an assumption, all the wireless nodes are identical; they are equipped as the FD system and have equal initial transmit power. Besides, the node combination in a single BRF transmission is randomly selected in the network. In the traffic model, we assume the transmission is the single relaying transmission flow with a relay node, and all transmitting nodes always have a DATA frame of the same size. This paper considers only one transmission per DATA frame for theoretical study purposes without a retransmission scenario. Therefore, the backoff timer is set to the initial backoff contention window for any collision. With the log-distance pathloss model as the channel model, the transmission rate is computed based on the SINR model, and link capacity model. And then, the numerical analysis of the proposed OATC scheme, RTS/FCTS, and FD-MCST is conducted for performance evaluation. The simulation results are based on an average of 1,000 different experiments. In the output model, we consider the performance matrices of achievable network capacity, average achievable throughput, and average achievable transmission overhead with the SINR threshold of 6 dB.

### 4.2. Evaluation Metrics

This section provides the evaluation metrics of the proposed OATC scheme in terms of achievable network capacity, achievable throughput, and achievable transmission overhead ratio.

#### 4.2.1. Achievable Network Capacity

The achievable network capacity, $C_{a}$ is the total of the achievable transmission capacity of every single BRF transmission in the network and computed as

(10) $$C_{a}=\sum_{m=1}^{M}C_{m}$$

where $C_{m}$ is the achievable transmission capacity, *C* of *m*th BRF transmission.

#### 4.2.2. Achievable Throughput

The two-state Markov Chain model, i.e., success and collision, as in [1] is applied to evaluate the proposed OATC scheme. A transmission collision will happen when a transmitting node starts transmission in a backoff slot, and one or more other stations start transmission in the same backoff slot. In the discrete-time Markov Chain, the next state of the transmission depends on the previous state, and the transmission collision occurs according to the conditional collision probability (*p*) of a transmission which is computed as

(11) $$p=\frac{2p^{\prime }}{1+p^{\prime }}$$

where $p^{\prime }$ is the probability that the transmitting node observes the collision.

By simply considering the FD system in a saturated traffic condition with no retransmission and the MAC protocol with the specification of IEEE802.11, the calculations for conditional collision probability and saturation throughput are similar as in [29]. Therefore, the saturation throughput, *S* of the transmission, is given as

(12) $$S=p_{t}\cdot \frac{(1-p)L}{(1-p)L+pC}\cdot \frac{1}{L}$$

where $p_{t}$ is the probability of transmission, L and C are the successful transmission time and transmission collision time, respectively.

The average achievable throughput, $S_{a}$ is the average value of the total number of successfully transferred data in a second by a transmitting node in the network and is formulated as

(13) $$S_{a}=\frac{p_{s}}{M}\sum_{m=1}^{M}S_{m}$$

where $p_{s}$ is the probability of successful transmission based on the SINR constraint condition of the transmission and $S_{m}$ is the saturation throughput, *S* of *m*th BRF transmission.

#### 4.2.3. Achievable Transmission Overhead

Suppose that the transmission is successful with the schematic analysis of frame exchange of CT scheme and MCST scheme as in [29]. We suppose that $T_{DIFS}$ is the duration of DCF interframe space (DIFS), $T_{SIFS}$ is the duration of short interframe space (SIFS), $T_{RTS}$ is the duration for request-to-send (RTS) frame, $T_{CTS}$ is the duration for clear-to-send (CTS) frame, $T_{ACK}$ is the duration for acknowledgement (ACK) frame, $T_{PHY}$ is the time of physical preamble and header, *H* is the size of MAC header and trailer, and $R_{base}$ is the basic rate. Therefore, the overhead time $T_{o}$ for CT scheme and MCST scheme defined by $T_{o}^{CT}$ and $T_{o}^{MCST}$, respectively, are

(14) $$\begin{array}{cc} \; & T_{o}^{CT}=T_{DIFS}+T_{RTS}+T_{CTS}+T_{PHY}+\frac{H}{R_{base}}+T_{ACK}+4T_{SIFS}\\ \; & T_{o}^{MCST}=T_{DIFS}+T_{RTS}+T_{STS}+T_{PHY}+\frac{H}{R_{base}}+T_{ACK}+4T_{SIFS} \end{array}$$

And then, the successful time of transmission to transmit an *l*-byte DATA length, $T_{s}$ for CT scheme and MCST scheme defined by $T_{s}^{CT}$ and $T_{s}^{MCST}$, respectively, are

(15) $$\begin{array}{cc} \; & T_{s}^{CT}=T_{BK}+T_{o}^{CT}+\frac{l}{\min\{R_{SR}^{con},R_{RD}^{con}\}}\\ \; & T_{s}^{MCST}=T_{BK}+T_{o}^{MCST}+\frac{l}{R^{MCST}} \end{array}$$

where $T_{BK}$ is the time of the initial backoff timer for the DCF mechanism and $R^{MCST}$ is the transmission rates of SR transmission and RD transmission, which is given as

(16) $$R^{MCST}=\left\{\begin{array}{c} \tau_{SR}\cdot R_{SR}^{con}+\left(1-\tau_{SR}\right)\cdot R_{SR}^{seq}\;\mathrm{if}\;SR\;\mathrm{is}\;\mathrm{sequential}\;\mathrm{transmission}\\ \tau_{RD}\cdot R_{RD}^{con}+\left(1-\tau_{RD}\right)\cdot R_{RD}^{seq}\;\mathrm{if}\;RD\;\mathrm{is}\;\mathrm{sequential}\;\mathrm{transmission} \end{array}\right.$$

On the other hand, by assuming the collision is detected during the initial stage of data transmission in the DCF mechanism, the time of transmission in the collision condition, $T_{c}$ is computed as

(17) $$T_{c}=T_{BK}+T_{RTS}+DIFS$$

The average achievable transmission overhead, $O_{a}$ is the average value of the overhead which is required to successfully carry out a transmission without considering the retransmission for any collision scenario in the network and is computed as

(18) $$O_{a}=\frac{1}{M}\sum_{m=1}^{M}O_{m}\times 100\%$$

where $O_{m}$ is the transmission overhead ratio, *O* of *m*th BRF transmission, which is given as

(19) $$O=\frac{T_{o}}{T_{s}}$$

### 4.3. Simulation Results and Discussion

**Figure 4:**
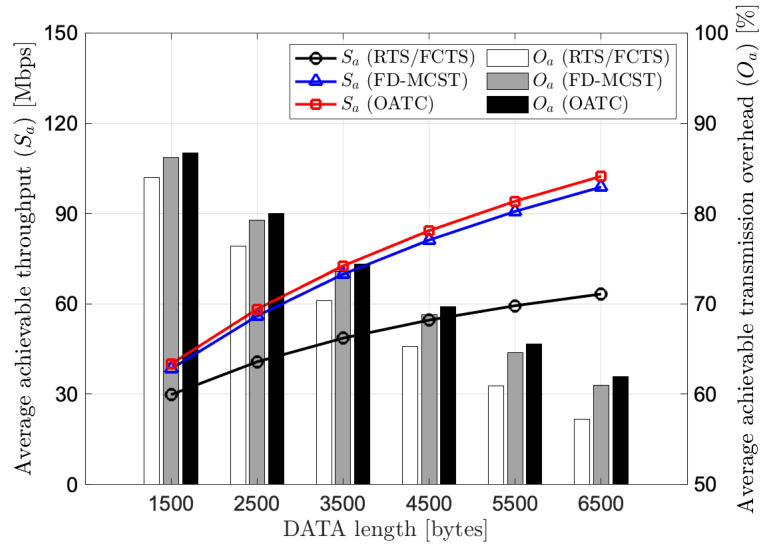
Performance of average achievable throughput and average achievable transmission overhead versus DATA length for 30-node random topology.

Figure 4 compares the performance of average achievable throughput and average achievable transmission overhead versus DATA length for the 30-node random topology scenario. As we can observe, OATC accomplishes its superiority in achievable throughput over RTS/FCTS and FD-DMAC regardless of the DATA length. Quantitatively, when the DATA length is 1500 bytes, OATC gives 40.02 Mbps, while RTS/FCTS and FD-MCST bring 29.84 Mbps and 38.42 Mbps, respectively. It means OATC accomplishes 34% and 4% of achievable throughput improvement compared to RTS/FCTS and FD-MCST, respectively. This is because OATC optimizes the achievable transmission capacity by considering the tradeoff threshold with the temporal and spatial reuse mechanisms. Compared to the other two protocols, when the DATA length increases, the achievement of OATC in average achievable throughput increases with high average achievable transmission overhead. However, the optimal achievable transmission capacity leads to a shorter successful transmission time and results a higher achievable transmission overhead. We can conclude that OATC is superior to the achievable throughput with the reasonably achievable transmission overhead regardless of the number of BRF transmissions and DATA length in the network.

Table 2 shows the performance comparison between the proposed OATC scheme, RTS/FCTS, and FD-MCST in terms of achievable network capacity and average achievable throughput for the network with single BRF transmission and fixed DATA length of 1500 bytes. The simulation result shows that there is no interference in the network with single BRF transmission. As we can observe, OATC achieves a better achievable network capacity and average achievable throughput by taking advantage of higher achievable transmission capacity through the MCST scheme. And then, Table 3 shows the performance of RTS/FCTS and FD-MCST with and without CTPC compared to OATC. With the fixed DATA length of 1500 bytes, we can observe that the CTPC scheme can achieve higher average achievable throughput to both the RTS/FCST and FD-MCST. However, by taking the advantage of temporal reuse and spatial reuse and considering the tradeoff threshold between the interference power and capacity gain, OATC can accomplish higher average achievable throughput compared to RTS/FCTS and FD-MCST.

**Figure 5:**
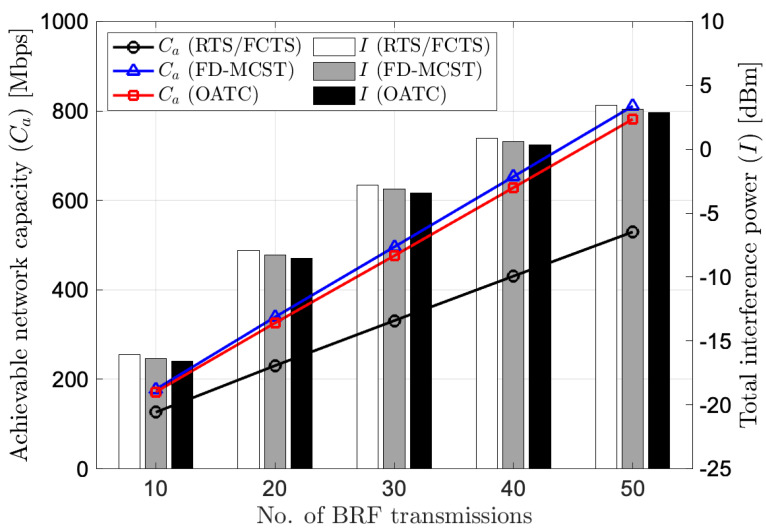
Performance of achievable network capacity and total interference power versus number of BRF transmissions in the network.

Figure 5 describes the performance comparison of achievable network capacity and total interference power versus the number of BRF transmissions in the multihop wireless network environments. As we can observe from the graph, OATC gives a better achievable network capacity compared to RTS/FCTS. In particular, OATC achieves 781.63 Mbps while RTS/FCTS achieves 529.45 Mbps when the number of BRF transmissions is 50. OATC can bring the 36% to 48% of better achievable network capacity given by RTS/FCTS when the number of BRF transmissions changes from 10 to 50 in the network. However, when the network is dense, OATC has a lower achievable network capacity than the FD-MCST. Quantitatively, OATC has around 4% lower achievable network capacity given by FD-MCST when the number of BRF transmissions is 50, which is neglectful compared to the achievement over RTS/FCTS. This is because OATC controls the transmit power with the CTPC scheme with the consensus coefficient to mitigate the interference power. Besides, the algorithms of OATC choose the operational transmission scheme subject to the minimum total interference power. In addition, Figure 5 depicts that the total interference power increases when the network density is high. The comparison shows that the OATC is superior to RTS/FCTS and FD-MCST in total interference power. We can conclude that OATC can accomplish a higher achievable network capacity and mitigate the interference power regardless of the number of BRF transmissions and density of the network.

**Figure 6:**
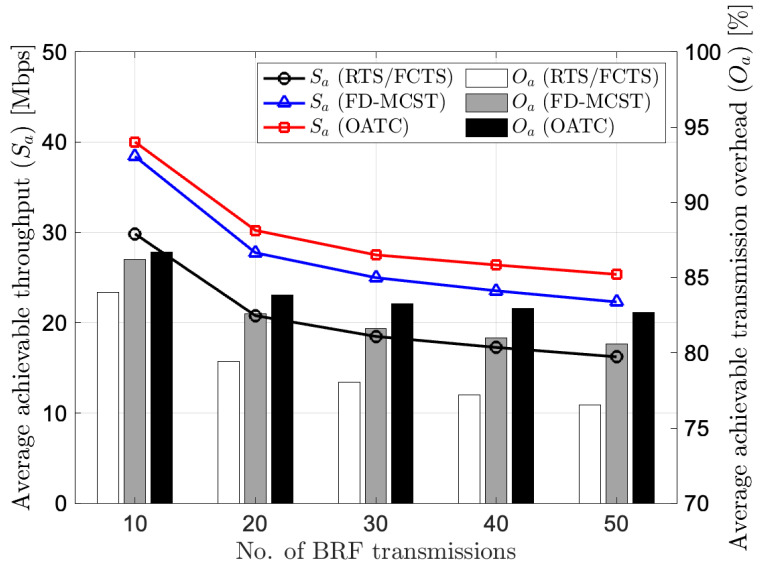
Performance of average achievable throughput and average achievable transmission overhead versus number of BRF transmissions in the network.

Figure 6 illustrates the performance comparison in terms of average achievable throughput and average achievable transmission overhead versus the number of BRF transmissions. OATC depicts the superiority of average achievable throughput compared to RTS/FCTS and FD-MCST with a fixed DATA length of 1500 bytes. This is because OATC optimizes the transmit power with the CTPC scheme and mitigates the interference power while satisfying the SINR constraint of the transmission. As a result, OATC converges the transmission rate of ongoing transmissions and maximizes the number of successful transmissions. The figure shows that the average achievable throughput decreases when the number of BRF transmissions increases. This is because the average transmitter-to-receiver distance decreases in the fixed network coverage area; on the other way, the network becomes dense. However, regardless of the number of BRF transmissions, OATC achieves the maximum achievable throughput over the other two protocols. Besides, the figure shows that the average achievable transmission overhead of OATC is higher than RTS/FCTS and FD-MCST, significantly when the number of BRF transmissions increases. In particular, OATC gives the average achievable overhead ratio of 83% while RTS/FCTS and FD-MCST give 77% and 81%, respectively, when the number of BRF transmissions is 50. This is because the higher transmission rate achieved by OATC gives a shorter successful transmission time of transmission compared to RTS/FCTS and FD-MCST.

Table 4 lists the performance analysis of concurrent transmissions in the OATC scheme. When the number of BRF transmissions increases, the network becomes dense, leading to high total interference. As we can observe, the ratio of the CT scheme operating as the operational transmission scheme in OATC is decreasing to mitigate the interference because of the concurrent transmissions. As a result, reducing the CT scheme in OATC increases the achievable network capacity and average achievable throughput gain. Furthermore, it decreases total interference power compared to the purely CT scheme, i.e., RTS/FCTS, when the number of BRF transmissions increases. However, the total interference reduction becomes merely less when the number of BRF transmissions is 50 due to the selection of BRF transmission that leads to the close distance between source and destination. In summary, the proposed OATC accomplishes its superiority in terms of achievable network capacity, total interference power, and achievable throughput compared to FD MAC protocol with RTS/FCTS and FD-MCST in multihop wireless networks.

## 5. Conclusions

This paper proposes an OATC scheme for capacity optimization in FD multihop wireless networks with the relaying system. We investigated the capacity optimization in terms of achievable network capacity, achievable throughput, and interference mitigation compared to the existing FD MAC protocol with RTS/FCTS and FD-MCST. Numerical simulation results reveal that only a concurrent transmission scheme and a mixture of concurrent and sequential transmission scheme do not guarantee the optimal transmission capacity in the FD system. Therefore, it is crucial to consider the sequential transmission and the aid of transmit power control scheme because of the interference. By combining the concurrent transmission scheme and a mixture of concurrent and sequential transmission scheme, the proposed OATC scheme optimizes the achievable network capacity with the tradeoff between the interference power and the received power of the transmission. Compared to the FD MAC with only concurrent transmissions, the simulation results show that the proposed OATC scheme is superior in interference mitigation and significantly accomplishes a higher achievable network capacity. In addition, the proposed OATC outperforms the other protocols in achievable throughput regardless of the number of BRF transmissions and DATA length. Mainly, OATC can accomplish 56% and 14% higher achievable throughput of RTS/FCTS and FD-MCST, respectively, in the FD multihop wireless network. As the preliminary research study, this paper only considers the FD system with a single relaying transmission flow with a relay node. The proposed OATC scheme can be easily extended for the wireless network with multiple relay nodes as well as the relaying selection, which is left as a further research topic for capacity optimization. The significant research attention on the FD system for capacity optimization is timely and ongoing in the trend of future 6G mobile communication systems. In future research work, we will conduct the performance investigation of the OATC scheme in multihop wireless networks with multiple relay nodes and highly dynamic environments such as vehicular scenarios. Furthermore, we will investigate OATC with the multi-channel allocation scheme for additional interference mitigation and capacity optimization in the FD multihop wireless networks.

## Figures and Tables

**Table 1:** Simulation Parameters and Settings.

Parameter	Value
Network coverage size	100 m × 100 m
Transmit power (*P*)	20 dBm
Propagation model	Log-distance pathloss
Attenuation constant (α)	3.5
Wall attenuation ($W_{ij}$)	0 dB
Shadowing parameter ($X_{\sigma }$)	8 dB
Noise level (η)	−174 dBm
Channel bandwidth (*B*)	20 MHz
Basic rate ($R_{base}$)	24 Mbps
PHY header duration	44 μs
MAC header	320 bits
RTS size	160 bits
CTS size	112 bits
STS size	120 bits
ACK size	112 bits
DIFS length	34 μs
SIFS length	16 μs
DATA length (*l*)	1500, 2500, 3500, 4500, 5500, 6500 bytes
Contention window (CW)	16 Slot time
Slot time length (σ)	9 μs
Consensus coefficient (C)	0.5∼3
Number of simulations	1000 times

**Table 2:** Performance Comparison between RTS/FCTS, FD-MCST, and OATC for the network with single BRF transmission and a fixed DATA length of 1500 bytes.

	Achievable Network Capacity [Mbps]	Average Achievable Throughput [Mbps]
RTS/FCTS	77.07	150.04
FD-MCST	78.47	195.92
OATC	78.47	195.92

**Table 3:** Performance Comparison between RTS/FCTS, FD-MCST with and without TPC mechanism and OATC for the network with a fixed DATA length of 1500 bytes.

	Average Achievable Throughput [Mbps]				
**No. of BRF** **Transmissions**	**RTS/FCTS**		**FD-MCST**		**OATC**
	**No TPC**	**TPC**	**No TPC**	**TPC**	
10	29.84	31.78	38.42	39.68	40.02
20	20.79	23.52	27.75	29.99	30.23
30	18.48	21.34	24.99	27.28	27.51
40	17.27	20.45	23.54	26.17	26.40
50	16.22	19.49	22.30	25.13	25.36

**Table 4:** Performance analysis of concurrent transmissions in OATC.

No. of BRF Transmissions	Ratio of CT in OATC	Interference Reduction [dBm]	$C_{a}$ Gain	$S_{a}$ Gain
10	0.11	0.53 (3%)	36%	34%
20	0.06	0.62 (8%)	41%	45%
30	0.05	0.61 (22%)	44%	49%
40	0.04	0.54 (61%)	46%	53%
50	0.03	0.60 (17%)	48%	56%

## Data Availability

Not applicable.

## Abbreviations

The following abbreviations are used in this manuscript:

BRF	Basic relaying flow transmission
CSMA/CA	Carrier sense multiple access with collision avoidance
CT	Concurrent transmission scheme
CTPC	Consensus transmit power control scheme
DCF	Distributed coordination function
FD	Full-duplex
FD-MCST	FD MAC protocol with MCST scheme
HD	Half-duplex
IUI	Inter-user interference
MAC	Medium access control protocol
MCST	Mixture of concurrent and sequential transmission scheme
OATC	Optimal achievable transmission capacity scheme
RD	Relay-destination transmission
RTS/CTS	Request-to-send/clear-to-send mechanism
RTS/FCTS	Request-to-send/full-duplex clear-to-send MAC protocol
SI	Self-interference
SIC	Self-interference cancellation
SINR	Signal-to-interference-plus-noise ratio
SNR	Signal-to-noise ratio
SR	Source-relay transmission
TPC	Transmit power control scheme
